# Genetic Polymorphism and Population Genetic Structure Analysis of 21 Autosomal STR Loci for a Han-Chinese Population from Luzhou of Southwest China

**DOI:** 10.3390/genes14071419

**Published:** 2023-07-09

**Authors:** Binghui Song, Jiewen Fu, Jie Qian, Lisha Yang, Jingliang Cheng, Junjiang Fu

**Affiliations:** 1 Key Laboratory of Epigenetics and Oncology, The Research Center for Preclinical Medicine, Southwest Medical University, Luzhou 646000, China; songbinghui@stu.swmu.edu.cn (B.S.); jingliangc@swmu.edu.cn (J.C.); fujiewen@swmu.edu.cn (J.F.); 20210199120039@stu.swmu.edu.cn (J.Q.); yls1988314@163.com (L.Y.); 2 Laboratory of Forensic DNA, The Judicial Authentication Center, Southwest Medical University, Luzhou 646000, China; 3 Basic Medical School, Southwest Medical University, Luzhou 646000, China; 4 Department of Obstetrics and Center for Prenatal Diagnosis, The Affiliated Hospital of Southwest Medical University, Luzhou, 646000, China

**Keywords:** STR, genetic polymorphism, Luzhou Han-Chinese population, forensic genetics, population data

## Abstract

The Han nationality is an ancient and populous people, and different places in China may have their distinct group relationships. Luzhou area, as a crossroads of several provinces in Southwest China, lacks autosomal short tandem repeat (STR) research and population genetics research. In this study, 21 autosomal STR loci were evaluated in 1959 Han-Chinese individuals from Luzhou area. There was no substantial linkage disequilibrium (LD) among the 21 autosomal STR markers, and all markers were in Hardy–Weinberg equilibrium (HWE). The total discrimination power (TDP) and cumulative probability of exclusion (CPE) of the 21 autosomal STR loci were calculated to be 1–9.8505 × 10^−16^ and 1–1.9406 × 10^−9^, respectively. There were 333 alleles for 21 STRs with allelic frequencies ranging from 0.00026 to 0.51302, and the number of alleles ranged from 7 in locus TPOX to 29 in locus Penta E. According to the results of population comparison and population differentiation, historical influences, geographical distribution, cultural integration, and economic development may have an impact on the Luzhou Han population and other Chinese populations. These 21 STR loci were found to enrich autosomal STR information in forensic databases and provide highly informative polymorphisms for our forensic practice in China, including personal identification and parentage testing.

## 1. Introduction

Short tandem repeat (STR) typing is the current standard approach for forensic DNA analysis, which involves polymerase chain reaction (PCR) amplification of selected STRs in the DNA template and capillary electrophoresis (CE) separation of the PCR amplification products [1,2]. The autosomal STR system is used in forensic DNA analysis to authenticate kinship, identify the deceased or missing person, and link the suspect to the crime site [3,4]. In forensic medicine and population genetics, DNA analysis based on highly polymorphic autosomal STR loci has been most commonly used [5]. To supply more genetic information and raise the discriminating power (DP) and probability of exclusion (PE), more STR markers with high genetic polymorphisms are added to amplification systems. Therefore, the study of allele distribution, genetic polymorphism, and related forensic parameters of autosomal STR multiplex amplification system in the target population can assure the scientific and accurate identification results of forensic genetics.

The STR online allele frequency database has now been developed with the endorsement of the International Society of Forensic Genetics (ISFG), and centralized quality control and data management are essential to maintain the high quality standards required for forensic genetics [6]. STR typing data in India [7] and Pakistan [8] can determine the genetic relationship of populations to other Asian populations. In addition, the STR marker datasets obtained in this study provides a valuable source of information for STR profiling of personnel, which can be used for disaster victim identification in military emergencies and added to the Indian army database and military hospital repository [9]. A large number of A-STR studies are also being conducted in Southeast Asian countries such as Vietnam, Thailand, and Malaysia [10,11,12], thereby revealing the close genetic relationship between mainland Southeast Asian populations and Southern Chinese populations, as well as improving the reliability and efficiency of DNA analysis in criminal cases and parentage testing [13]. The above studies have made considerable progress in multi-population and multi-ethnic areas. In the Americas, the wave of research on STR polymorphisms of Native Americans not only enriched the STR database, but also provided data information on the geographical distribution of tribes and genetic differences among tribal populations, contributing to the development of research on the geographic isolation and genetic variation of Native American tribes [14,15,16]. More importantly, the study of allele and genotype frequencies is an essential prerequisite for the use of any human polymorphic genetic markers in forensic work. Therefore, forensic geneticists need to make an effort to investigate and report genetic marker data for relevant populations. Moreover, the number of alleles, the frequency of allele distribution and other related forensic parameters of the autosomal STR locus vary across populations, so the diversity of populations and the available genetic markers have become a hot topic for forensic research.

Luzhou is the meeting point of Sichuan Province, Guizhou Province, Yunnan Province, and Chongqing City in the geographical center areas of Southwest China. Luzhou City is also located in southeast Sichuan Province, upstream of the Yangtze River, in the transition zone between the southern margin of Sichuan Basin and Yunnan-Guizhou Plateau, with geographical coordinates of 27°39′ to 29°20′ north latitude and 105°8′ to 106°28′ east longitude. Luzhou has a permanent population of 4,254,149 people, according to China’s Seventh National Census Bulletin, with a growth rate of 0.85 percent (http://tjj.luzhou.gov.cn/tjsj/tjgb/content_802318, accessed on 20 March 2022). Since the Qin Dynasty, Luzhou has had multiple population migrations, the most renowned of which was the “Huguang Tian Sichuan” Immigration Movement during the Qing Dynasty. The Han ethnic group is the most populous in this area, and ethnic minorities such as the Miao, Yi, Hui, and others are widespread. For nearly five years, genetic diversity researches of autosomal STR markers were reported in Han-Chinese groups from Yunnan Province [17], Sichuan Province [18], Chongqing City [19], and Guizhou Province [20] in southwestern China. Genetic polymorphism observed from 21 non-combined DNA index system (CODIS) STR loci in the Chengdu Han population with 210 individuals were reported in 2018. Moreover, the mutation study of autosomal STR markers and Y-STR markers in the Han ethnic group from Southwest China was also reported [21,22,23]. In the Luzhou area, the Han population and various ethnic groups have formed a unique group relationship, and its genetic characteristics and population structure have not been thoroughly studied. However, not only the allele frequency for autosomal STR markers in the Luzhou Han population but also the genetic relationships and population structure of the Luzhou Han population and other Han-Chinese populations and ethnic minorities in China are unknown. In this study, we evaluated 21 autosomal STR loci in 1959 Han-Chinese individuals from Luzhou area; we also collected 21 Han-Chinese groups and 12 ethnic minority groups in China to reveal the substructure and diversity among Chinese groups including Luzhou Han. Therefore, it is necessary to study the genetic polymorphisms and forensic application value for autosomal STRs of the Han population in the Luzhou area from Southwest China and explore germline development and genetic differentiation in the Chinese population.

## 2. Materials and Methods

### 2.1. Regents

The AGCU Expressmarker 22 kit (EX22 kit) was purchased from AGCU ScienTech Incorporation, Wuxi, China to study the Han population in the Luzhou area, which included 21 autosomal STR loci and 1 sex-determining locus, namely, D2S441, D2S1338, D3S1358, D5S818, D6S1043, D7S820, D8S1179, D10S1248, D12S391, D13S317, D16S539, D18S51, D19S433, D21S11, Penta E, TPOX, TH01, CSF1PO, Penta D, vWA, FGA, and amelogenin. Chelex-100 was purchased from Bio-Rad Laboratories Co., Ltd., Hercules, CA, USA (Cat. #: 143-2832).

### 2.2. Samples Collection and Genomic DNA Extraction

With informed consent, we collected blood samples from 1959 unrelated healthy individuals (1262 males and 697 females) of the Han ethnic group in the Luzhou area. We used the Chelex-100 protocol to extract genomic DNA from the FTA card (Shandong Chengwu Ronghua Biotechnology Co., Ltd., Heze, China) [24]. A 1 mm diameter blood spot sample was taken on an FTA card using a punch and added to a 600 µL centrifuge tube. Then, 400 µL of double distilled water (ddH_2_O) was added for shaking and left at room temperature for 30 min. The tubes were centrifuged for 3 min at 12,000 r on a high-speed centrifuge (Thermo Fisher, Waltham, MA, USA); then, the supernatant was removed and 5% Chelex-100 35 µL was added to it. The tubes were placed in a constant temperature water bath at 56 °C for 30 min, followed by shaking for 5 s and a boiling water bath for 8 min; then, centrifuged at 12,000 r for 3 min after 5 s of shaking. The 20 µL of supernatant was taken into a new 600 µL centrifuge tube for subsequent polymerase chain reaction (PCR) amplification. For long-term storage, the samples were stored in the refrigerator at −20 °C.

### 2.3. PCR Amplification and Autosomal STR Genotyping

The 21 autosomal STR markers were amplified using the EX22 kit system according to the manufacturer’s instructions. PCR amplification was generated in a machine for Thermal Cycler (Applied Biosystems, Life Technology, Waltham, MA, USA). For further information, a 10 μL PCR reaction volume containing Reaction Mix 4.0 μL, EX22 Primers 2.0 μL, C-Taq Polymerase 0.4 μL, genomic DNA sample 3 μL, and sterile deionized water 0.6 μL was used for each sample. The PCR program was set to pre-denaturation at 95 °C for 2 min; denaturation at 94 °C for 30 s, annealing at 60 °C for 1 min, extension at 72 °C for 1 min, cycle number 10; denaturation at 90 °C for 30 s, annealing at 58 °C for 1 min, extension at 72 °C for 1 min, cycle number 20; and final extension at 72 °C for 10 min, maintenance at 4 °C. After PCR amplification, 3 μL of product was mixed with 10 μL sample mixture, including AGCU Marker SIZE-500 30 μL and deionized formamide 1000 μL. The 3500 Dx Genetic Analyzer (Applied Biosystems, Life Technology, Waltham, MA, USA) was applied to separate the PCR products by capillary electrophoresis and GeneMapper ID-X software (Thermo Fisher, Waltham, MA, USA) was used to analyze the results [25]. For quality, control DNA 9947A was also supplied in this kit. The laboratory has been authenticated by the China National Accreditation Service for Conformity Assessment (CNAS) and Accreditation Criteria for the Competence of Testing and Calibration Laboratories (ISO/IEC 17025:2017). The certification and accreditation of CNAS indicated that the laboratory had the technical ability to conduct testing and calibration services in accordance with the corresponding accreditation criteria. As a specific external quality assurance program, proficiency testing of CNAS includes parentage testing of trios, parentage testing of duos, individual identification, full sibling testing, etc. The autosomal STR genotype analysis was carried out in accordance with the Specification of Parentage Testing by China (GB/T 37223-2018). The guidelines for the population study of STR from ISFG have been followed [26]. In addition, all methods were performed in accordance with the relevant guidelines and regulations.

### 2.4. Data Analysis

The allele frequencies, Hardy–Weinberg equilibrium (HWE), and corresponding forensic parameters including typical paternity index (TPI), polymorphism information content (PIC), matching probability (MP), and so on were evaluated by Modifed-PowerStats software. The test of LD and the population differentiation analysis between the target population and previously published relevant population data were analyzed by the Arlequin v3.5 software [27]. The autosomal STR information form 33 relevant Chinese populations, including two Manchu, two Mongolian, two Hui, two Yi, one Kazakh, one Uyghur, one Li, one Korean, and 21 Han populations [19,20,28,29,30,31,32,33,34,35,36,37,38,39,40,41], were collected from previously published studies to explore genetic similarities and divergences. In particular, the Sichuan Han sample is from Chengdu, Sichuan Province. The R project software (version 4.0.5) (https://www.r-project.org/, accessed on 3 May 2021) was used to build a map displaying the approximate geographic positions of these populations. The Nei’s standard genetic distance between the Luzhou Han ethnic group and other 33 relevant Chinese populations was calculated by a modified PHYLIP program [42]. And the heatmap of standard genetic distance between these above-mentioned populations was plotted by OriginPro software [43]. According to the normalized allele frequencies of 19 autosomal STR markers in 34 populations, principal component analysis (PCA) was evaluated by Multivariate Statistical Package (MVSP) software. Based on the standard genetic distance, multidimensional scale (MDS) and neighbor-joining (NJ) phylogenetic trees were implemented in IBM SPSS 25 and MEGA-X software [44], respectively.

## 3. Results

### 3.1. Linkage Disequilibrium, Allele Frequencies, and Forensic Parameters

In this study, the Luzhou Han population was tested for linkage disequilibrium (LD) using autosomal STR genotypes from 1959 samples. After Bonferroni correction (*p* = 0.05/210 ≈ 0.00024), no significant LD was found among the 21 autosomal STR loci (Table S1), manifesting that these loci were statistically independent. The allelic frequencies and forensic parameters including *p*-values of exact tests for HWE of 21 autosomal STRs were presented in Table S2 and Table 1, respectively. The results found that all the 21 STR markers were demonstrated to obey HWE after Bonferroni correction (*p* = 0.05/21 ≈ 0.00238). The total discrimination power (TDP) and the cumulative probability of exclusion (CPE) of the 21 autosomal STRs were calculated using the genetic data and were found to be 1–9.8505 × 10^−16^ and 1–1.9406 × 10^−9^, respectively.

The corresponding allelic frequencies ranged from 0.00026 to 0.51302 in 333 alleles across 21 STRs, with the number of alleles ranging from 7 in locus TPOX to 29 in locus Penta E (Table S2). In addition, the largest number of genotypes was 171 in locus Penta E, and the smallest number was 16 in locus TPOX. In the 21 autosomal STR loci, locus Penta E had the highest polymorphism information content (PIC) as 0.90394, and locus TPOX had the lowest PIC as 0.55691. We also calculated the averages of forensic parameters including DP of 0.79585, PE of 0.59580, MP of 0.07527, PIC of 0.76716, TPI of 2.74650, expected heterozygosity (H_exp_) of 0.79605, and observed heterozygosity (H_obs_) of 0.79465 in these STRs. Except for locus TPOX and TH01, the values of H_obs_ and DP were all greater than 0.7 in 19 of 21 autosomal STR loci. Moreover, the greatest values of H_obs_ and DP were observed in locus Penta E as 0.90812 and 0.91048, respectively.

### 3.2. Population Structure and Population Comparisons

**Figure 1:**
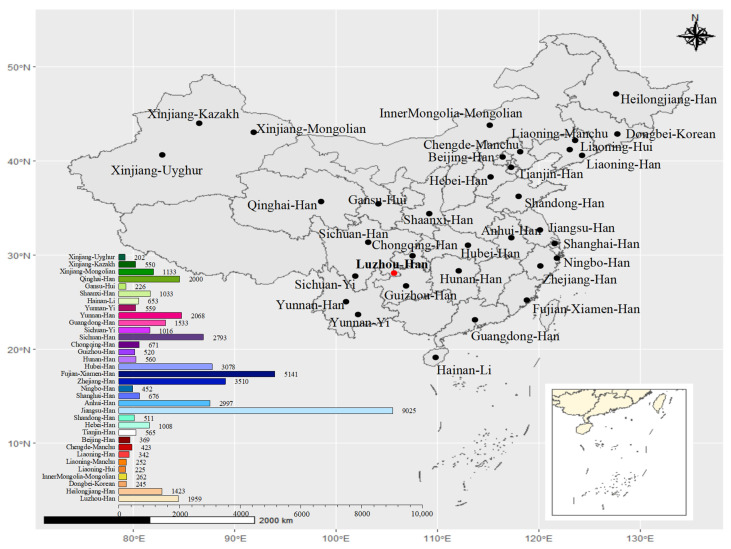
Map of Han population in the Luzhou area and populations from other areas in China. Map showing the approximate geographic positions of the studied Han population in the Luzhou area of Southwest China and other 33 reference populations. The number of samples in different populations is presented at the left-bottom of the figure. The R project software (version 4.0.5) (https://www.r-project.org/, accessed on 3 May 2021) and DATAV GeoAtlas (areas_v3) (https://datav.aliyun.com/tools/atlas/index.html, accessed on 3 May 2021) were used to create this map. The circle “●” in red represents the Luzhou area. The circle “●” in black represents the other 33 areas.

**Figure 2:**
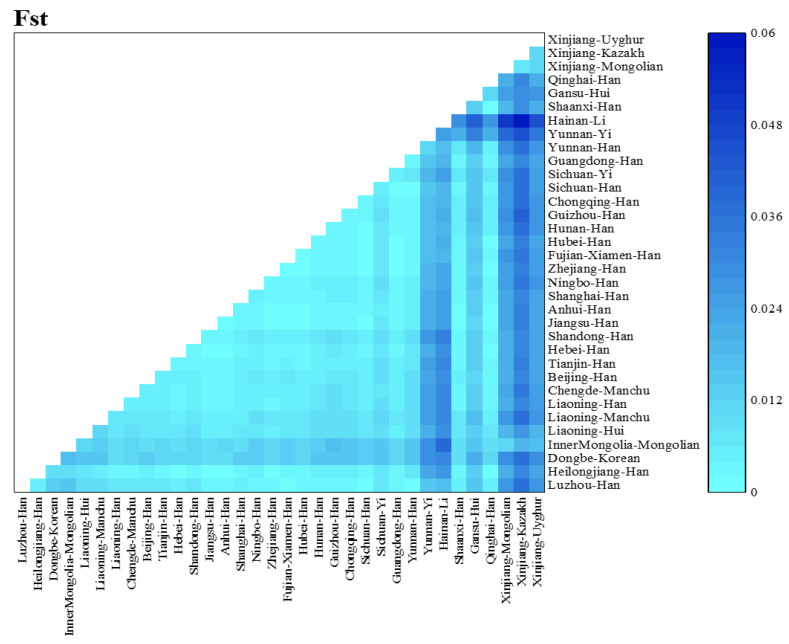
Heatmap of the standard genetic distance of the Luzhou Han population and the other 33 reference populations. The heatmap was plotted by OriginPro software version 9.9.5 (https://www.originlab.com/, accessed on 16 February 2022).

**Figure 3:**
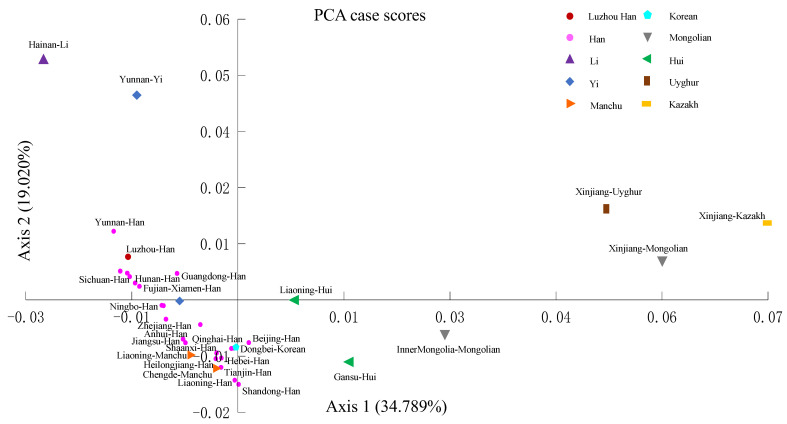
Map for principal component analysis (PCA) from the 34 Chinese populations. PCA was estimated based on the allele frequencies of 19 autosomal STR loci in the 34 Chinese populations.

Nei’s standard genetic distance matrix was used to compare the Luzhou Han population to the other 33 Chinese populations based on the frequency of alleles at 19 autosomal STR loci (Table S3). Figure 1 depicted the approximate geographic positions of these populations in China, as well as their corresponding sample numbers. From Table S3, the results presented that the Sichuan Han was the nearest population to the Luzhou Han with a genetic distance of 0.00105, followed by the Yunnan Han (0.00174), the Fujian Xiamen Han (0.00195), the Guangdong Han (0.00204), the Hubei Han (0.00226), the Zhejiang Han (0.00238), the Guizhou Han (0.00266), the Hunan Han (0.00279), and the Chongqing Han (0.00286). The farthest genetic distance to the Luzhou Han was the Xinjiang Kazakh (0.03699), followed by the Xinjiang Mongolian (0.02711) and the Xinjiang Uyghur (0.02630). Both the Hainan Li, the Xinjiang Uyghur, the Xinjiang Mongolian, and the Xinjiang Kazakh share large genetic distance values with other groups. In addition, the distance matrix was visualized using a heatmap of standard genetic distance between these populations (Figure 2). Furthermore, we used the normalized allele frequencies of 19 autosomal STR loci in 34 populations to perform PCA (Figure 3). In this plot, the first and second principal component could explain 34.789% and 19.020% of the total variance, respectively. We noticed a clear population stratification between these populations, roughly divided into three segments. The ethnic minorities in northern China were scattered on the right side of the plot, including the Liaoning Hui, the Gansu Hui, the Inner Mongolia Mongolian, the Xinjiang Uyghur, the Xinjiang Mongolian, and the Xinjiang Kazakh, while the ethnic minorities in southern China were mainly distributed on the upper left of the plot, including the Hainan Li and the Yunnan Yi. Additionally, although Han populations were gathered in the lower left of the plot, the southern Han populations and the northern Han populations were divided into two clusters in this segment. It is interesting to note that certain ethnic minorities and their geographically similar Han nationality were closely distributed in the PCA plot, such as the Chengde Manchu and the Hebei Han, suggesting the integration of diverse ethnic groups.

**Figure 4:**
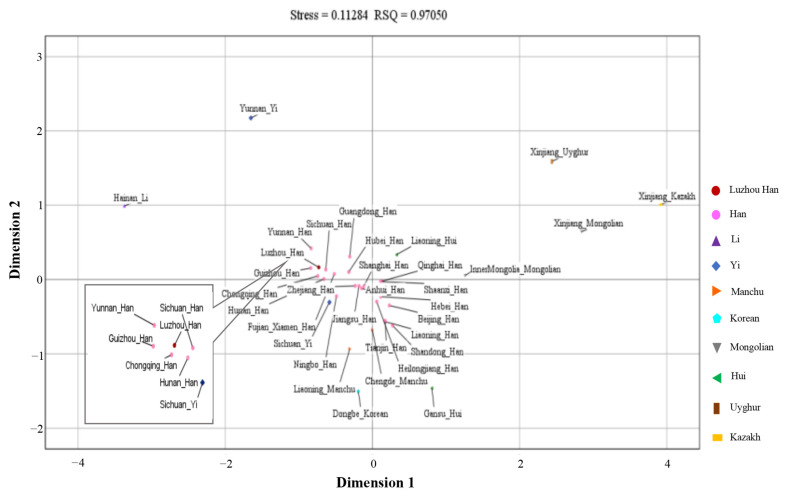
Multidimensional scaling (MDS) plot results. MDS plot displays the genetic relationships between the Luzhou Han and the other 33 reference populations in China.

**Figure 5:**
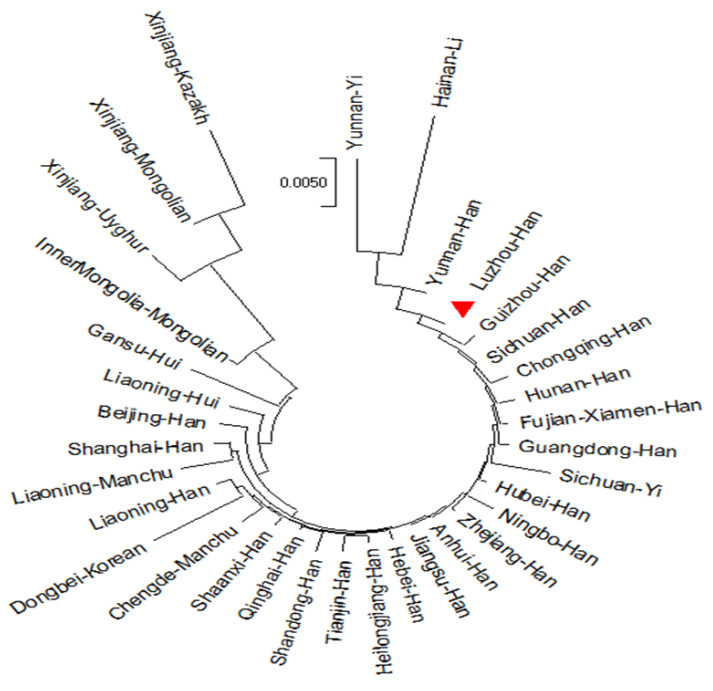
The phylogenetic tree between the Luzhou Han population and the other 33 reference populations. The phylogenetic tree displays the genetic relationships between the Luzhou Han population and the other 33 reference populations. The phylogenetic tree was constructed using the neighbor-joining method of MEGA-X software version 10.0.5 (https://www.megasoftware.net/, accessed on 16 February 2022).

According to the MDS plot based on the Euclidean distance model (Figure 4), we discovered that all Han nationality groups were concentrated in the middle of the plot, and many minorities were scattered around the plot. Furthermore, some minorities were closely located with Han populations, such as the Sichuan Yi, the Liaoning Hui, and the Chengde Manchu. In the lower left of the plot, the enlarged image showed that Luzhou Han was close to the Chongqing Han, the Sichuan Han, the Guizhou Han, the Hunan Han, and the Yunnan Han. Some minorities in the plot were far apart from the other populations, including the Yunnan Yi, the Hainan Li, the Xinjiang Kazakh, the Xinjiang Uyghur, and the Xinjiang Mongolian. The NJ phylogenetic tree was constructed from the Nei’s standard distance matrix (Figure 5), which showed evolutionary history and relatedness among these populations. It revealed a clear distinction between northern and southern Chinese groups. Among them, some northern ethnic minorities were quite different from other groups and formed their unique clusters, such as the Gansu Hui, the Inner Mongolia Mongolian, the Xinjiang Uyghur, the Xinjiang Mongolian, and the Xinjiang Kazakh. The Luzhou Han was relatively close to the Guizhou Han, the Yunnan Han, the Sichuan Han, the Chongqing Han, and the Hunan Han, and formed a cluster. Many populations formed the same cluster with groups from adjacent areas, including the Ningbo Han and the Zhejiang Han, the Jiangsu Han and the Anhui Han, the Liaoning Han and the Dongbei Korean. In addition, the Hainan Li and the Yunnan Yi showed a relatively distant relationship with southern Chinese groups and formed a unique cluster. Thus, these results proved that historical influences, geographical distribution, cultural integration, and economic development could have an impact on the studied majority groups.

In Table S4, we performed population comparisons by calculating the Fst values and their relevant *p* values between the studied Luzhou Han ethnic group and the other 33 reference Chinese populations at 19 autosomal STR loci. The Xinjiang Mongolian and the Xinjiang Kazakh presented significant genetic differences with the Luzhou Han at 11 and 9 STR loci, respectively. The calculation showed that the Yunnan Yi was the only population with statistically significant differences from the Luzhou Han at three STR loci, followed by the Hainan Li at two STR loci. In addition, the Heilongjiang Han, the Dongbei Korean, the Hebei Han, the Jiangsu Han, the Guizhou Han, the Sichuan Yi, and the Xinjiang Uyghur owned significant differences with the Luzhou Han at one locus after Bonferroni correction (*p* = 0.05/627 ≈ 0.00008). However, there were no statistically significant differentiations between the Luzhou Han and many other populations in any of these STR markers, including the Inner Mongolia Mongolian, the Liaoning Hui, the Liaoning Manchu, the Liaoning Han, the Chengde Manchu, the Fujian Xiamen Han, the Beijing Han, the Tianjin Han, the Shandong Han, the Anhui Han, the Shanghai Han, the Ningbo Han, the Zhejiang Han, the Hubei Han, the Hunan Han, the Chongqing Han, the Sichuan Han, the Guangdong Han, the Yunnan Han, the Shananxi Han, the Gansu Hui, and the Qinghai Han.

## 4. Discussion

The Han nationality is an ancient and populous ethnic group that has developed its distinct group relations in different regions of China. According to China’s Seventh National Census Bulletin, the southwest Han population accounts for around 12.78% of the total Han population in China. Southwest China includes Chongqing City, Sichuan Province, Guizhou Province, Yunnan Province, and Tibet Autonomous Region, among which the Sichuan Basin is the region’s most densely populated, conveniently accessible, and economically developed area. The Luzhou area is the meeting point of Sichuan Province, Guizhou Province, Yunnan Province, and Chongqing City and the transition zone between the southern margin of Sichuan Basin and Yunnan–Guizhou Plateau, which formed a unique group relationship, and its genetic characteristics and population structure have not been thoroughly studied. There are certain regional differences in STR allele among Han populations in various provinces of China, and preliminary biogeographic inference of individual origin can be made based on the results of STR typing and the frequency of alleles, while the accuracy of regional inference can be significantly improved by increasing the number of STR loci. In addition, due to the scattered nature and limitations of population genetics studies and the large differences in sample sizes of Han and ethnic minority populations in different geographical areas, more abundant data should be obtained. Despite belonging to the same ethnic group, groups located in different geographical areas may have different subpopulation structures and genetic characteristics. Therefore, population genetic analysis of the Luzhou Han can not only confirm the forensic application value of genetic markers in this population, but also provide a corresponding genetic basis for the study of ethnic origin, formation, and development [45] and genetic relationship.

In this study, by recruiting 1959 unrelated healthy Han-Chinese individuals, the TDP and CPE of the 21 autosomal STRs were calculated using the genetic data and were found to be 1–9.8505 × 10^−16^ and 1–1.9406 × 10^−9^, respectively. Because some of the 21 STR loci are located on the same chromosome, such as TPOX, D2S441 and D2S1338, D5S818 and CSF1PO, D21S11 and Penta D, and vWA and D12S391, we performed an LD test. It could show that these autosomal STR loci can be used to calculate the combined parentage index (CPI) and can be applied in this population’s forensic practice. After Bonferroni correction, the 21 autosomal STRs all showed no significant LD and observed HWE. If 0.05 is used as a small probability in multiple comparisons, it will increase the probability of occurrence of Type I error. There were 210 comparisons among 21 loci after pairwise comparison, and the probability of Type I error was high, which did not meet the criteria of small probability judgment [46]. Although Bonferroni correction is relatively conservative, it is still commonly used in forensic genetics. Recent studies have shown that Bonferroni correction applied to HWE tests of multiple loci in forensic population genetics is detrimental to the discovery of deviation loci [47]. In this study, we noted that only the D2S441 locus owned a significant HWE departure when using an unadjusted α. Table 1 showed that the actual observed genotype number of the D2S441 locus is 50, which is much lower than the expected genotype number inferred from the allele number, i.e., 153. In addition, it owned more rare alleles. This departure from HWE may be caused by individual migration, genetic exchange, or gene mutation. The corresponding allelic frequencies ranged from 0.00026 to 0.51302 in 333 alleles across 21 STRs, and the averages of forensic parameters included DP of 0.79585, PE of 0.59580, expected heterozygosity (H_exp_) of 0.79605, and observed heterozygosity (H_obs_) of 0.79465 in these STRs. The 21 autosomal STR markers could provide highly informative polymorphisms for our forensic practice in China, including personal identification and parentage testing.

The Nei’s standard genetic distance matrix showed that the Luzhou Han population was close to Sichuan Han (0.00105) among the other 33 Chinese populations, followed by the Yunnan Han (0.00174), the Fujian Xiamen Han (0.00195), the Guangdong Han (0.00204), the Hubei Han (0.00226), the Zhejiang Han (0.00238), the Guizhou Han (0.00266), the Hunan Han (0.00279), and the Chongqing Han (0.00286). In addition, the Beijing Han (0.00752) have the furthest genetic distance in the Han-Chinese population (diversity), while the Sichuan Yi (0.00655) and the Chengde Manchu (0.00719) are somewhat closer with the Luzhou Han. It is worth noting that the Luzhou Han, the Guizhou Han, the Chongqing Han, the Sichuan Han, the Hunan Han, and the Guangdong Han were closely distributed in the PCA plot. The Dongbei Korean and the Chengde Manchu are closely distributed with the Han-Chinese population (admixture), and most of the Han-Chinese populations are close to adjacent Han-Chinese groups. Furthermore, the MDS plot and the NJ phylogenetic tree showed a similar population genetic structure and population relationships. We can find that Han-Chinese populations have significant population differentiation and genetic differences from most of China’s ethnic minorities, such as the Kazakh, Mongolian, Uyghur, Hui, Li and Yi, and the Han-Chinese groups in different provinces have a distant genetic relationship consistent with their geographical distribution (diversity). Interestingly, some ethnic minority groups such as the Chengde Manchu and the Dongbei Korean may be integrated with the neighboring Han-Chinese and are more closely related genetically (admixture). According to the Fst values and their relevant *p* values between the studied Luzhou Han ethnic group and the other 33 reference Chinese populations, the Xinjiang Mongolian, the Xinjiang Kazakh, the Yunnan Yi, the Hainan Li, the Heilongjiang Han, the Dongbei Korean, the Hebei Han, the Jiangsu Han, the Guizhou Han, the Sichuan Yi, and the Xinjiang Uyghur owned significant differences with the Luzhou Han. Historical influences, geographical distribution, cultural integration, and economic development may have an impact on the population’s genetic structure and diversification of ethnic groups. More importantly, we revealed the substructure and diversity of Chinese Han groups and minority groups, showing the homogeneity and difference between the Han-Chinese population and the integration of minority and neighboring Han groups.

In conclusion, this study was the first to explore allele frequencies and corresponding forensic parameters of these STR loci in Luzhou Han of Southwest China, as well as the population genetic structure between the studied group and other Chinese groups. We proved that these 21 STR loci could enrich autosomal STR information in the forensic databases and provide highly informative polymorphisms for our forensic practice in China, including personal identification and parentage testing. The research might not only fill the gap of autosomal STR research and population genetic differentiation in the center junction areas of Southwest China but also reveal substructure, diversity, and admixture in the Chinese population. In addition, the research also explores the relationship between the Luzhou Han population and other Chinese groups, which can play a fundamental role in the research fields of human genetics, forensic medicine, linguistics, molecular anthropology, and archaeology.

## Figures and Tables

**Table 1:** The corresponding forensic statistical parameters for 21 autosomal STR loci of Han population in Luzhou area, Southwest China (*n* = 1959).

Loci	Allele Number	Genotype Number	H_exp_	H_obs_	MP	DP	PIC	PE	TPI	H	h	*p*
D3S1358	10	30	0.72390	0.73813	0.12732	0.72372	0.67410	0.48968	1.90936	0.26187	0.73813	0.15893
D13S317	10	33	0.79143	0.78765	0.07384	0.79122	0.76060	0.57632	2.35457	0.21235	0.78765	0.68060
D7S820	17	41	0.76764	0.75447	0.08543	0.76745	0.73458	0.51744	2.03638	0.24553	0.75447	0.16729
D16S539	10	34	0.77706	0.76059	0.08369	0.77686	0.74158	0.52806	2.08849	0.23941	0.76059	0.07989
Penta E	29	171	0.91071	0.90812	0.01489	0.91048	0.90394	0.81203	5.44167	0.09188	0.90812	0.68739
D2S441	17	50	0.78755	0.76161	0.07683	0.78735	0.75646	0.52984	2.09743	0.23839	0.76161	0.00501
TPOX	7	16	0.62104	0.62379	0.20662	0.62088	0.55691	0.32040	1.32904	0.37621	0.62379	0.80216
TH01	17	41	0.67218	0.67841	0.15619	0.67201	0.62663	0.39565	1.55476	0.32159	0.67841	0.55690
D2S1338	15	74	0.86107	0.85503	0.03448	0.86085	0.84570	0.70480	3.44894	0.14497	0.85503	0.43972
CSF1PO	13	32	0.72433	0.71261	0.11938	0.72414	0.67852	0.44803	1.73979	0.28739	0.71261	0.24061
Penta D	13	49	0.80353	0.80194	0.06140	0.80333	0.77977	0.60265	2.52448	0.19806	0.80194	0.85929
D10S1248	11	39	0.75110	0.76110	0.10113	0.75091	0.71309	0.52895	2.09295	0.23890	0.76110	0.30603
D19S433	19	79	0.82256	0.83206	0.05407	0.82235	0.80055	0.65982	2.97720	0.16794	0.83206	0.27134
vWA	12	35	0.80033	0.81113	0.07149	0.80013	0.77039	0.61985	2.64730	0.18887	0.81113	0.23203
D21S11	26	101	0.81959	0.81674	0.05348	0.81938	0.79758	0.63048	2.72841	0.18326	0.81674	0.74339
D18S51	19	94	0.86284	0.86269	0.03449	0.86262	0.84788	0.72001	3.64126	0.13731	0.86269	0.98448
D6S1043	22	95	0.87351	0.88821	0.02993	0.87329	0.85994	0.77140	4.47260	0.11179	0.88821	0.05030
D8S1179	13	49	0.84916	0.83512	0.04099	0.84894	0.83044	0.66576	3.03251	0.16488	0.83512	0.08255
D5S818	12	34	0.78003	0.76927	0.08223	0.77983	0.74657	0.54331	2.16704	0.23073	0.76927	0.25014
D12S391	15	65	0.84844	0.85860	0.04194	0.84823	0.83019	0.71189	3.53610	0.14140	0.85860	0.20985
FGA	26	114	0.86904	0.87034	0.03071	0.86882	0.85515	0.73533	3.85630	0.12966	0.87034	0.86458

H_exp_: expected heterozygosity; H_obs_: observed heterozygosity; MP: matching probability; DP: discrimination power; PIC: polymorphism information content; PE: probability of exclusion; TPI: typical paternity index; H: Homozygotes; h: Heterozygotes; *p*: *p* value of the exact test in Hardy–Weinberg equilibrium.

## Data Availability

All data used for the analyses in this report are available from the corresponding author upon reasonable request.

## Supplementary Materials

The following supporting information can be downloaded at: https://www.mdpi.com/article/10.3390/genes14071419/s1, Table S1: *p*-values of linkage disequilibrium test for all pairs of 21 STR loci in Han population in Luzhou area; Table S2: Allele frequencies for 21 autosomal STR loci of Han population in Luzhou area, Southwest China (*n* = 1959); Table S3: Genetic distances between the Luzhou Han population and other 33 relative Chinese reference populations; Table S4: Pairwise Fst and corresponding *p*-values of population comparisons between the Luzhou Han population and 33 reference populations.
